# Factors associated with early inhospital adverse outcome following surgery for acute appendicitis in Uganda: a multicenter cohort

**DOI:** 10.1186/s13741-024-00412-9

**Published:** 2024-06-03

**Authors:** Sharif Yusuf Farhan, Demoz Abraha, Isaac Edyedu, Selamo Fabrice Molen, William Mauricio, Samuel Oledo Odong, Michael Mugeni, Joshua Muhumuza

**Affiliations:** Department of Surgery, Faculty of Clinical Medicine and Dentistry, Kampala International University Western Campus, Ishaka-Bushenyi, Uganda

**Keywords:** Acute appendicitis, Inhospital early adverse outcomes, Factors, Uganda

## Abstract

**Introduction:**

Surgery for acute appendicitis has been associated with significant morbidity. This study aimed to determine the factors associated with early inhospital adverse outcomes following surgery for acute appendicitis in Uganda.

**Methods:**

This was a multicentre, prospective cohort in which early inhospital outcome following surgery for acute appendicitis was assessed at 4 regional referral hospitals in Uganda. The occurrence of complications during the admission period was documented as well as the length of hospital stay. Factors associated with adverse outcomes were determined using Poisson regression.

**Results:**

Of the 102 patients who underwent surgery for acute appendicitis, the majority were males 79(77.5%) with a mean age of 23.8(SD = 12.5) years. The perforated appendix was seen in 26 (25.5%) patients. Post-operative complications occurred in 21(20.6%) with the commonest being surgical site infection in 19(18.6%) patients. The median length of hospital stay was 3(IQR = 3–4) days with 43(42.2%) staying in hospital for more than 3 days. The presence of anemia (Hb < 8) (aRR = 1.376, CI = 1.159–1.634, *P* =  < 0.001) and having a perforated appendix (aRR = 1.263, CI = 1.026–1.555, *P* = 0.027) were independently associated with occurrence of complications while being HIV positive (aRR = 1.379, CI = 1.105–1.721, *P* = 0.005) and having a perforated appendix (aRR = 1.258, CI = 1.019–1.554, *P* = 0.033) were independently associated with prolonged hospital stay.

**Conclusion:**

Community education about early presentation is still required in order to reduce the number of patients that present late which should, in turn, reduce the risk of complications and length of hospital stay.

**Supplementary Information:**

The online version contains supplementary material available at 10.1186/s13741-024-00412-9.

## Background

Acute appendicitis is one of the most common causes of acute abdominal pain that requires surgical treatment (Bhangu et al. 2020), with a lifetime risk of 8.6% in males and 6.7% in females (Snyder et al. 2018). It can have a number of etiologies, all of which can result in intraluminal bacteria invading the appendicular wall (Carr 2000) and is primarily diagnosed clinically. Patients with suspected acute appendicitis may also be evaluated using ultrasonography, computed tomography (CT), or magnetic resonance imaging (MRI) (Snyder et al. 2018). About 80% of instances of appendicitis are uncomplicated (also known as simple appendicitis), which is typically treated with an immediate appendectomy (Talan and Saverio 2021). The most serious complication is perforation, which can result in abscesses, peritonitis, problems with fertility, and sepsis. Even with improved imaging, perforation rates range from 17 to 32%, which may result in a longer hospital stay, longer antibiotic use, and other more serious postoperative consequences (Snyder et al. 2018).

At Nsambya Hospital in Uganda, perforated appendix was one of the most common causes of peritonitis (Ojuka et al. 2014). The annual healthcare cost for appendicitis-related hospitalizations was calculated to be $3 billion according to a global systematic review done in 2017 (Ferris et al. 2017).

Though appendicitis has been associated with significant morbidity following surgery in other countries with better health structures (Talan and Saverio 2021; Rahman et al. 2020), the outcomes in Uganda are not known but are expected to be different since the type of care offered has been shown to affect early outcomes (Yuan et al. 2022). For example, laparoscopic surgery has been shown to reduce complications (Biondi et al. 2016), yet laparoscopic appendectomy is not available in most facilities in Uganda. This study aimed to determine the factors associated with early inhospital adverse outcomes following surgery for acute appendicitis at four selected regional referral hospitals in Uganda.

## Methods

### Study design and period

This was a multicentre, prospective cohort done in the surgery departments of four regional referral hospitals; specifically Hoima, Jinja, Mubende, and Fortportal, over a period of 3 months (June–August 2023).

### Study setting

The 4 hospitals are located in four different regions of Uganda and each hospital has general surgeons and senior house officers in the surgery departments. These doctors are responsible for the care of patients with acute appendicitis. On average, each hospital receives 10 patients with acute appendicitis per month. At these hospitals, if surgery is indicated, patients undergo open appendectomy.

### Study population

Patients presenting with acute appendicitis at the selected study centers during the study period were included in the study if they consented. Patients who presented with an appendicular mass (phlegmon) or abscess were excluded since these should be managed non-operatively, while those with appendicular abscess require only a drainage procedure.

### Sample size and sampling

A formula for sample size with finite population correlation by Daniel was used to calculate the sample size.$n=\frac{ N{z}^{2}P(1-P)}{{d}^{2}\left(N-1\right)+{z}^{2}P(1-P)}$. P = 0.5 (representative proportion in the study population), *N* = 120 (expected number of patients with acute appendicitis at the study centers over a 3 months period), *Z* = 1.96 (*Z* value statistic at 95% confidence level) *d* = 0.05 (the acceptable error). Substituting into the formula, *n* = 92. On adding 10% to cater for loss of follow-up and increase internal validity, the required sample size was 102. Consecutive sampling was done till the sample size was reached.

### Recruitment and data collection procedure

Any patient confirmed to have acute appendicitis was informed about the role of the study and asked to participate. Those that accepted to participate, signed an informed consent document. For consented patients, a pretested data collection tool was filled to obtain the data on the study variables. A clinical examination was done and a sample was drawn for a complete blood count. An ultrasound scan was also done to rule out appendicular mass and abscess. The patients with a diagnosis of acute appendicitis underwent surgery as appropriate. Postoperatively, patients were assessed daily to assess for the occurrence of complications until discharge.

### Study variables

The dependent variable was early inhospital adverse outcomes following surgery. The adverse outcomes included the occurrence of complications and hospital stay greater than the median. The independent variables included social-demographic characteristics (age, sex, residence, education, and smoking) and clinical characteristics (duration of symptoms, Alvarado score, clinical pattern, comorbidity, hemodynamic status, hemoglobin, and white blood cell count). The clinical pattern referred to whether the appendix was perforated or if it was uncomplicated/simple appendicitis. Hemodynamic instability referred to a combination of hypotension and tachycardia. Anemia referred to a hemoglobin of less than 8 g/dl.

### Quality control

The data collection tool was pretested for validity and reliability and adjustments were made before starting data collection. The research protocol was explained to the research assistants during training. Every day, the principal investigator or his assistant double-checked the data to make sure it was accurate. A general surgeon supervised the research and all procedures were done by a qualified surgeon or a senior house officer under the supervision of a general surgeon.

### Data analysis

The data obtained was summarized in Excel sheets using Microsoft Office Excel (Microsoft Corporation, Washington, USA, version 13.0 for Windows). The summarized data was imported into Statistical Package for Social Sciences (SPSS Inc., Chicago, USA, version 26.0 for Windows). The percentage of the specific outcomes was computed as a fraction of all the patients managed for acute appendicitis. The corresponding 95% confidence intervals were reported. The median length of hospital stay and the corresponding interquartile ranges were also computed since the length of hospital stay was not normally distributed. Patients with either surgical site infection or any other complication were considered to have early inhospital adverse outcomes. Bivariate and multivariate analysis was done using Poisson regression reporting both risk ratios and *P* values. Variables with *P* value ≤ 0.2 at bivariate were reanalyzed at multivariate using Poisson regression. Variables with *P* ≤ 0.05 were considered significant factors associated with early inhospital adverse outcomes. The length of hospital stay was transformed into a categorical variable using visual binning at the median, and the factors associated with the length of hospital stay above the median were determined using Poisson regression.

### Ethical considerations and consent

All methods were carried out in accordance with relevant guidelines and regulations. Ethical approval was granted by the Research and Ethics Committee of Bishop Stuart University (Ref No: BSU-REC-2023–117). All participants gave written informed consent as evidenced by the participants’/guardian’s signature.

## Results

Of the 102 patients who underwent surgery for acute appendicitis in this study, the majority were males 79(77.5%) with a mean age of 23.8(SD = 12.5) years. Most were from rural areas 74(72.5%) with a history of smoking reported among 19(18.6%). Twenty-six (25.5%) had a perforated appendix while the remaining 76 (74.5%) had uncomplicated appendicitis. In this study, 21 out of 102 patients got one or more complications before discharge, representing a complication rate of 20.6%. The commonest complication was surgical site infection seen in 19(18.6%) of the study participants. The median length of hospital stay was 3(IQR = 3–4) days with 43(42.2%) staying in hospital for 4 days or more. None of the patients died. The rest of the details on adverse outcomes are shown in Table 1 below.

**Table 1:** Adverse outcomes following surgery for acute appendicitis

Outcome	Frequency	Percentage
Complications		
No complication	81	79.4 (CI = 71.6–87.3)
One or more complications	21	20.6 (CI = 12.7–28.4)
*SSI*	*19*	*18.6*
*Hemorrhage*	*1*	*1.0*
*Other*	*3*	*2.9*
Length of hospital stay (days)	Median = 3, IQR = 3–4, Min = 1, Max = 12	
≤ Median (≤ 3 days)	59	57.8
> Median (4 + days)	43	42.2

*SSI* surgical site infection, *IQR* interquartile range, *Min* minimum, *Max* maximum

Regarding factors associated with early inhospital complications, at the bivariate level of analysis, the variables that had a *p* value less than 0.2 and, therefore, qualified for multivariate analysis were age greater than 45 years, presence of hypertension, duration of symptoms greater than 3 days at presentation, Alvarado score of 7–10, presence of anemia and having a perforated appendix. The detailed bivariate analysis is shown in Supplementary file 1: Table S1. At the multivariate level of analysis, the presence of anemia (Hb < 8) (aRR = 1.376, CI = 1.159–1.634, *P* =  < 0.001) and having a perforated appendix (aRR = 1.263, CI = 1.026–1.555, *P* = 0.027) were independently associated with the occurrence of complications. The risk of having complications was increased by 1.376 times if the patient had anemia (Hb < 8) and by 1.263 times if the patient had a perforated appendix. The rest of the multivariate analysis findings are shown in Table 2.

**Table 2:** Analysis of factors associated with the occurrence of complications

Characteristic	Bivariate analysis			Multivariate analysis		
	cRR	95% CI	*P* value	aRR	95% CI	*P* value
Age (years)						
6–17 (children)	Ref					
18–45 (adults)	2.240	0.587–8.542	0.238	1.093	0.138–8.684	0.933
46–61 (older adults)	4.571	0.821–25.465	0.083	1.147	0.044–30.016	0.935
Hypertension						
No	Ref					
Yes	3.208	0.659–15.615	0.149	1.168	0.604–2.259	0.644
Symptoms (days)						
≤ Median (3)	Ref					
4 +	1.312	1.128–1.526	< 0.001	1.130	0.958–1.333	0.146
Alvarado score						
5–6	Ref					
7–10	1.141	0.972–1.339	0.107	0.941	0.793–1.118	0.492
Anemia (Hb < 8)						
No	Ref					
Yes	**1.477**	**1.257**–**1.735**	** < 0.001**	**1.376**	**1.159**–**1.634**	** < 0.001**
Pattern						
Simple	Ref					
Perforated	**1.484**	**1.210**–**1.820**	** < 0.001**	**1.263**	**1.026**–**1.555**	**0.027**

*aRR* adjusted risk ratio, *Ref* reference category, *CI* confidence interval

Regarding factors associated with increased hospital stay, at the bivariate level of analysis, the variables that had a *p* value less than 0.2 and therefore qualified for multivariate analysis were the presence of hypertension, diabetes or human immune deficiency virus, duration of symptoms greater than 3 days at presentation, hemodynamic instability at presentation and having a perforated appendix. The details of the findings of the bivariate analysis are shown in Supplementary file 1: Table S2. At the multivariate level of analysis, being HIV positive (aRR = 1.379, CI = 1.105–1.721, *P* = 0.005) and having a perforated appendix (aRR = 1.258, CI = 1.019–1.554, *P* = 0.033) were independently associated with increased hospital stay. An HIV-positive patient was 1.379 times more likely to have prolonged hospital stay while the risk was increased by 1.258 times in a patient who had a perforated appendix. The rest of the multivariate analysis findings are shown in Table 3.

**Table 3:** Analysis of factors associated with increased hospital stay

Characteristic	Bivariate analysis			Multivariate analysis		
	cRR	95% CI	*P* value	aRR	95% CI	*P* value
Hypertension						
No	Ref					
Yes	3.750	0.692**–**20.332	0.125	1.060	0.752**–**1.494	0.740
Diabetes						
No	Ref					
Yes	3.630	0.881**–**14.954	0.074	1.150	0.788**–**1.680	0.468
HIV						
Negative	Ref					
Positive	**4.167**	**1.209–14.359**	**0.024**	**1.379**	**1.105–1.721**	**0.005**
Symptoms (days)						
≤ Median (3)	Ref					
4 +	3.378	1.483**–**7.692	0.004	1.203	0.998**–**1.450	0.053
Instability (hypotension and tachycardia)						
No	Ref					
Yes	2.468	0.908**–**6.706	0.077	1.236	0.980**–**1.559	0.073
Pattern						
Simple	Ref					
Perforated	**3.632**	**1.424–9.268**	**0.007**	**1.258**	**1.019–1.554**	**0.033**

*aRR* adjusted risk ratio, *Ref* reference category, *CI* confidence interval

## Discussion

Majority of participants were males with a mean age of 23.8 years. This age distribution was expected since appendicitis is known to be common in the ages between 10 and 30 years (Nshuti et al. 2014). Also, appendicitis has been reported to be more common among males compared to females (Nshuti et al. 2014).

We noted that the majority of the participants had uncomplicated appendicitis. These findings are in agreement with a systematic review which reported the average rate of appendix perforation at presentation to be between 16 and 30% (Bhangu et al. 2020), as well as Snyder et al. (Snyder et al. 2018) who also reported that perforation rates among adults with acute appendicitis ranged from 17 to 32%. The proportion of patients with perforated appendix was lower than that reported in the USA (29.2%) (Prystowsky et al. (2005)) and Bangladesh (77.7%) (Rahman et al. 2020), but higher than that reported in Nigeria where 20% of the patients had a perforated appendix at presentation (Azodo and Omuemu 2017) as well as in Sudan where perforation rate was 23% (Azoz and Elhaj 2010). The high rate of perforated appendix seen in our study can be attributed to late presentation, given that the median time of presentation was 3 days with 48% of the participants presenting after 3 days of symptoms. The fact that the majority of participants came from rural areas could have contributed to the late presentation resulting in delayed treatment.

The commonest complication was surgical site infection seen in 19(18.6%) of the study participants. This was comparable to findings in Bangladesh where 19% got complications (Rahman et al. 2020) and another study which reported that surgical site infection was the commonest complication following surgery for acute appendicitis (Abass et al. 2021).

The complication rate in this study was lower than that reported in the Netherlands (25.0%) (16), Argentina among patients converted from laparoscopic to open (48.0%) (Monrabal Lezama et al. 2193), in Finland (24.4%) (Salminen et al. 2018) and in India 24% (Puthenvariath et al. 2020), but higher than that reported in the USA (10.6%) (Becker et al. 2019), in Israel (0.5%) (Tankel et al. 2020), in Italy (13.2%) (Minutolo et al. 2014), in Finland (4.8%) (Mönttinen et al. 2021), in Canada (3%) (Talan and Saverio 2021) and in Ethiopia (12%) (Ayele 2021).

The high rate of complications seen in our study can be attributed to the fact that a high percentage of the patients presented with a perforated appendix which would have increased the risk of complications. The rate of postoperative wound infection is determined by intraoperative wound contamination with rates of infection varying from < 5% in simple appendicitis to 20% in cases with perforation (Humes and Simpson 2006). Given that surgical site infection was the most common complication seen in this study, then the high rates of the perforated appendix could explain the high rates of complications. Also, some of the studies that reported a very low rate of complications had laparoscopic surgery which has been associated with a lower risk of complications, and this is possible because of patient selection where low-risk patients are operated on using the laparoscopic method.

The length of hospital stay in this study was comparable to that reported in Israel (mean = 2.4 days) (Tankel et al. 2020), Italy (mean = 3.87 days, range, 1–19) (Minutolo et al. 2014), and Nigeria where 47% were discharged in less than 3 days (Azodo and Omuemu 2017). The length of stay was lower than that reported in Argentina among patients converted from laparoscopic to open (mean = 5 days) (Monrabal Lezama et al. 2193), but higher than that reported in Sudan where 77% of the patients were discharged within 24 h (Abass et al. 2021). The high rate of complications arising from delayed presentation and perforation of the appendix could have contributed to the increased length of hospital stay.

It was noted that the presence of anemia and having a perforated appendix were independently associated with the occurrence of complications at the multivariate level of analysis. Our findings are in agreement with findings in Poland where complicated appendicitis was significantly associated with postoperative complications (Walędziak et al. 2019) and in the Netherlands where complications were more common in children with complex appendicitis (38%) and less in children with simple appendicitis (12%) (Knaapen et al. 2021). Perforation of the appendix increases contamination of the wound which increases the risk of surgical site infection; the commonest complication seen in this study. Also in line with our findings, one study (White et al. 2017) reported that anemia was associated with an increased risk of postoperative complications including surgical site infection. This is because anemia impairs the rate of wound healing which increases the chances of surgical site infection.

We also noted that being HIV positive and having a perforated appendix were independently associated with prolonged hospital stay. In agreement with our findings, a study in Poland reported that complicated appendicitis was significantly associated with prolonged length of hospital stay (Walędziak et al. 2019). This is possibly because rupture of the appendix increases the risk of complications which, in turn, increases the length of stay. Also in line with our findings, a study in Tanzania reported that HIV infection was common among patients with appendicitis and was associated with severe morbidity, postoperative complications, and longer hospital stays (Giiti et al. 2010). The association between HIV status and length of hospital stay is possibly because of its effect on all systems of the body which, in turn, delays recovery and discharge from hospital.

Though some studies have reported that extremes of age were associated with increased risk of adverse outcomes (Rahman et al. 2020; Yuan et al. 2022; Lasek et al. 2018), age was not a significant factor in our study possibly because we did not have anyone less than 5 years, nor did we have anyone above the age of 65 years, the age groups associated with an increased risk of adverse outcomes (Rahman et al. 2020; Yuan et al. 2022; Lasek et al. 2018).

### Strength and limitations

This was a multicentre study done in the different regions of Uganda which increases the generalizability of our findings. However, our study only assessed the factors listed under the study variables because they were thought to be the most relevant in our setting. Other factors not assessed in this study could also affect outcomes following surgery for acute appendicitis.

## Conclusion and recommendations

The proportion of patients with perforated appendixes was high. The proportion of patients with complications was high with a big proportion of patients spending more than 3 days in the hospital. The presence of anemia and having a perforated appendix were independently associated with the occurrence of complications while being HIV positive and having a perforated appendix were independently associated with prolonged hospital stay.

Education of the community about early presentation is still required in order to reduce the number of patients that present late, which will in turn reduce the risk of complications and length of hospital stay. This can be done through radio and televised programs educating the masses about the dangers associated with late presentation and how the risk of complications is increased when patients are delayed in reporting to the hospital.

When patients with anemia undergo an emergency appendectomy, they should be assessed closely to assess if there is an indication for transfusion in line with the relevant guidelines. If an indication for transfusion is found, then a blood transfusion should be done. In patients with no indication for blood transfusion, then other considerations like administration of hematinics should be considered.

### Supplementary Information

Supplementary Material 1: Supplementary Table S1. Bivariate analysis of factors associated with occurrence of complications. Supplementary Table S2. Bivariate analysis of factors associated with prolonged hospital stay.

## Data Availability

Data is available upon request. Requests should be sent to sharif.farhan@studwc.kiu.ac.ug (FYS).

## Abbreviations

cRR: Crude risk ratio
aRR: Adjusted risk ratio
CI: Confidence interval
