# Supplementary material for: Factors associated with early inhospital adverse outcome following surgery for acute appendicitis in Uganda: a multicenter cohort
Source: Perioper Med (Lond). 2024 Jun 3;13:51. doi: 10.1186/s13741-024-00412-9 (PMC11149220; doi:10.1186/s13741-024-00412-9)
Supplement: Supplementary file 1 — Supplementary Material 1: Supplementary Table S1. Bivariate analysis of factors associated with occurrence of complications. Supplementary Table S2. Bivariate analysis of factors associated with prolonged hospital stay. [file 13741_2024_412_MOESM1_ESM.docx]

**Table S1: Bivariate analysis of factors associated with occurrence of complications. more likely to have blood culture positive sepsiss (aOR=red patient who had a d the symptoms was**

| **Characteristic** | **No complications, N=81** | **Complications, N= 21** | **Bivariate analysis** | | |
| --- | --- | --- | --- | --- | --- |
|  |  |  | **cRR** | **95% CI** | **P value** |
| **Age(years)** |  |  |  |  |  |
| 6-17 (Children) | 24(23.5) | 3(2.9) | Ref |  |  |
| 18 - 45 (Adults) | 50(49.0) | 14(13.7) | 2.240 | 0.587-8.542 | 0.238 |
| 46-61(older Adults) | 7(6.9) | 4(3.9) | 4.571 | 0.821-25.465 | **0.083** |
| **Sex** |  |  |  |  |  |
| Male | 64(62.7) | 15(14.7) | Ref |  |  |
| Female | 17(16.7) | 6(5.9) | 1.506 | 0.508-4.467 | 0.461 |
| **Education** |  |  |  |  |  |
| None | 8(7.8) | 2(2.0) | 1.437 | 0.220-9.405 | 0.705 |
| Primary | 30(29.4) | 7(6.9) | 1.342 | 0.350-5.140 | 0.668 |
| Secondary | 20(19.6) | 8(7.8) | 2.300 | 0.601-8.797 | 0.224 |
| Tertiary | 23(22.5) | 4(3.9) | Ref |  |  |
| **Residence** |  |  |  |  |  |
| Rural | 58(56.9) | 16(15.7) | 1.269 | 0.416-3.867 | 0.675 |
| Urban | 23(22.5) | 5(4.9) | Ref |  |  |
| **Smoking** |  |  |  |  |  |
| No | 66(64.7) | 17(16.7) | Ref |  |  |
| Yes | 15(14.7) | 4(3.9) | 1.035 | 0.304-3.524 | 0.956 |
| **Hypertension** |  |  |  |  |  |
| No | 77(75.5) | 18(17.6) | Ref |  |  |
| Yes | 4(3.9) | 3(2.3) | 3.208 | 0.659-15.615 | **0.149** |
| **Diabetes** |  |  |  |  |  |
| No | 74(72.5) | 18(17.6) | Ref |  |  |
| Yes | 7(6.9) | 3(2.9) | 1.762 | 0.414-7.491 | 0.443 |
| **HIV** |  |  |  |  |  |
| Negative | 70(68.6) | 18(17.6) | Ref |  |  |
| Positive | 11(10.8) | 3(2.9) | 1.061 | 0.267-4.206 | 0.933 |
| **Symptoms(days)** |  |  |  |  |  |
| ≤ median (3) | 49(48.0) | 4(3.9) | Ref |  |  |
| 4+ | 32(31.4) | 17(16.7) | 1.312 | 1.128-1.526 | **<0.001** |
| **Instability (Hypotension and tachycardia)** | | |  |  |  |
| No | 66(64.7) | 16(15.7) | Ref |  |  |
| Yes | **15(14.7)** | 5(4.9) | 1.375 | 0.435-4.343 | 0.587 |
| **Alvarado score** |  |  |  |  |  |
| 5-6 | 18(17.6) | 2(2.0) | Ref |  |  |
| 7-10 | 63(61.8) | 19(18.6) | 1.141 | 0.972-1.339 | **0.107** |
| **Anemia (Hb<8)** |  |  |  |  |  |
| No | 58(56.9) | 3(2.9) | Ref |  |  |
| Yes | 23(22.5) | 18(17.6) | 1.477 | 1.257-1.735 | **<0.001** |
| **Leucocytosis** |  |  |  |  |  |
| No | 14(13.7) | 2(2.0) | Ref |  |  |
| Yes | 67(65.7) | 19(18.6) | 1.985 | 0.414-9.510 | 0.391 |
| **Pattern** |  |  |  |  |  |
| Simple | 68(66.7) | 8(7.8) | Ref |  |  |
| Perforated | 13(12.7) | 13(12.7) | 1.484 | 1.210-1.820 | **<0.001** |

*Ref= Reference category, cRR= Crude risk ratio, CI= Confidence interval,*

**Table S2: Bivariate analysis of factors associated with prolonged hospital stay. more likely to have blood culture positive sepsiss (aOR=red patient who had a d the symptoms was**

| **Characteristic** | **LOS ≤Medina (3), N=59** | **LOS>Medina (3), N= 43** | **Bivariate analysis** | | |
| --- | --- | --- | --- | --- | --- |
|  |  |  | **cRR** | **95% CI** | **P value** |
| **Age (years)** |  |  |  |  |  |
| 6-17 (Children) | 17(16.7) | 10(9.8) | Ref |  |  |
| 18 - 45 (Adults) | 37(36.3) | 27(26.5) | 1.241 | 0.492-3.129 | 0.648 |
| 46-61(older Adults) | 5(4.9) | 6(5.9) | 2.040 | 0.493-8.446 | 0.325 |
| **Sex** |  |  |  |  |  |
| Male | 43(42.2) | 36(35.3) | 1.914 | 0.709-5.163 | 0.200 |
| Female | 16(15.7) | 7(6.9) | Ref |  |  |
| **Education** |  |  |  |  |  |
| None | 6(5.9) | 4(3.9) | 0.533 | 0.122-2.332 | 0.404 |
| Primary | 27(26.5) | 10(9.8) | 0.296 | 0.104-1.847 | 0.223 |
| Secondary | 14(13.7) | 14(13.7) | 0.800 | 0.277-2.311 | 0.680 |
| Tertiary | 12(11.8) | 15(14.7) | Ref |  |  |
| **Residence** |  |  |  |  |  |
| Rural | 40(39.2) | 34(33.3) | 1.794 | 0.718-4.482 | 0.211 |
| Urban | 19(18.6) | 9(8.8) | Ref |  |  |
| **Smoking** |  |  |  |  |  |
| No | 50(49.0) | 33(32.4) | Ref |  |  |
| Yes | 9(8.8) | 10(9.8) | 1.684 | 0.618-4.586 | 0.308 |
| **Hypertension** |  |  |  |  |  |
| No | 57(55.9) | 38(37.3) | Ref |  |  |
| Yes | 2(2.0) | 5(4.9) | 3.750 | 0.692-20.332 | **0.125** |
| **Diabetes** |  |  |  |  |  |
| No | 56(54.9) | 36(35.3) | Ref |  |  |
| Yes | 3(2.9) | 7(6.9) | 3.630 | 0.881-14.954 | **0.074** |
| **HIV** |  |  |  |  |  |
| Negative | 55(53.9) | 33(32.4) | Ref |  |  |
| Positive | 4(3.9) | 10(9.8) | 4.167 | 1.209-14.359 | **0.024** |
| **Symptoms(days)** |  |  |  |  |  |
| ≤ median (3) | 38(37.3) | 15(14.7) | Ref |  |  |
| 4+ | 21(20.6) | 28(27.5) | 3.378 | 1.483-7.692 | **0.004** |
| **Instability (Hypotension and tachycardia)** | | |  |  |  |
| No | 51(50.0) | 31(30.4) | Ref |  |  |
| Yes | 8(7.8) | 12(11.8) | 2.468 | 0.908-6.706 | **0.077** |
| **Alvarado score** |  |  |  |  |  |
| 5-6 | 11(10.8) | 9(8.8) | Ref |  |  |
| 7-10 | 48(47.1) | 34(33.3) | 0.866 | 0.323-2.317 | 0.774 |
| **Anemia** |  |  |  |  |  |
| No | 35(34.3) | 26(25.5) | Ref |  |  |
| Yes | 24(23.5) | 17(16.7) | 0.954 | 0.428-2.127 | 0.907 |
| Leukocytosis |  |  |  |  |  |
| No | 10(9.8) | 6(5.9) | Ref |  |  |
| Yes | 49(48.0) | 37(36.3) | 1.259 | 0.420-3.775 | 0.682 |
| **Pattern** |  |  |  |  |  |
| Simple | 50(49.0) | 26(25.5) | Ref |  |  |
| Perforated | 9(8.8) | 17(16.7) | 3.632 | 1.424-9.268 | **0.007** |

*Ref= Reference category, cRR= Crude risk ratio, CI= Confidence interval, LOS=Length of hospital stay.*
